# Primary amelanotic malignant melanoma of the esophagus: a case report

**DOI:** 10.1186/s40792-019-0564-2

**Published:** 2019-01-11

**Authors:** Naomichi Koga, Nobuhide Kubo, Hiroshi Saeki, Shun Sasaki, Tomoko Jogo, Kosuke Hirose, Yuichiro Nakashima, Eiji Oki, Yutaka Koga, Yoshinao Oda, Hisao Oiwa, Toshio Oiwa, Yoshihiko Maehara

**Affiliations:** 1 Department of Surgery and Science, Graduate School of Medical Sciences, Kyushu University, 3-1-1, Maidashi, Higashi-ku, Fukuoka, 812-8582 Japan; 2 Department of Anatomic Pathology, Pathological Sciences, Graduate School of Medical Sciences, Kyushu University, 3-1-1, Maidashi, Higashi-ku, Fukuoka, 812-8582 Japan; 3 Oiwa Gastrointestinal Clinic, 2-1-5, Hanami-Higashi, Koga-shi, Fukuoka, 811-3112 Japan; 4 Kyushu Central Hospital of the Mutual Aid Association of Public School Teachers, 3-23-1, Shiobaru, Minami-ku, Fukuoka, 815-8588 Japan

**Keywords:** Esophagus, Amelanotic melanoma, Diagnosis, Prognosis, Pathology

## Abstract

**Background:**

Primary amelanotic malignant melanoma of esophagus, which is a subtype of primary malignant melanoma of the esophagus (PMME), is a very rare disease with a poor prognosis. We herein report a case of the amelanotic type of PMME.

**Case presentation:**

An 86-year-old woman was admitted to our hospital with symptoms of dysphagia. An endoscopic examination and constructed radiography revealed an elevated and semipedunculated lesion with an ulcer in the lower thoracic esophagus accompanied by another submucosal lesion of the esophagus. She was diagnosed with esophageal squamous cell carcinoma by a preoperative endoscopic biopsy. We performed thoracoscopy- and laparoscopy-assisted subtotal esophagectomy with lymphadenectomy. Based on the surgical specimens, although there were no melanocytes, we made a diagnosis of a malignant melanoma immunohistochemically; the tumor cells were positive for S-100 protein and HMB45 focally and partially for Melan-A.

**Conclusion:**

We experienced a case of primary amelanotic malignant melanoma, and the patient has remained disease-free for 1 year since the surgery. Since the diagnosis of amelanotic type of PMME is difficult, it should be made by the combination of a morphological examination, pathological examination, and immunohistochemistry.

## Background

Primary malignant melanoma of the esophagus (PMME) is a rare disease, accounting for 0.1–0.2% of malignant esophageal lesions [1]. Primary amelanotic malignant melanoma (amelanotic-type) accounts for 10–25% of all PMME, and its prognosis is extremely poor because of its highly malignant biological behavior and delays in the accurate diagnosis [2]. Although an endoscopic biopsy can aid in the diagnosis of PMME, its accuracy is unsatisfactory, especially for the diagnosis of amelanotic type [2]. There are few reports of amelanotic type PMME. We herein report an 86-year-old woman with amelanotic-type PMME.

## Case presentation

**Fig. 1:**
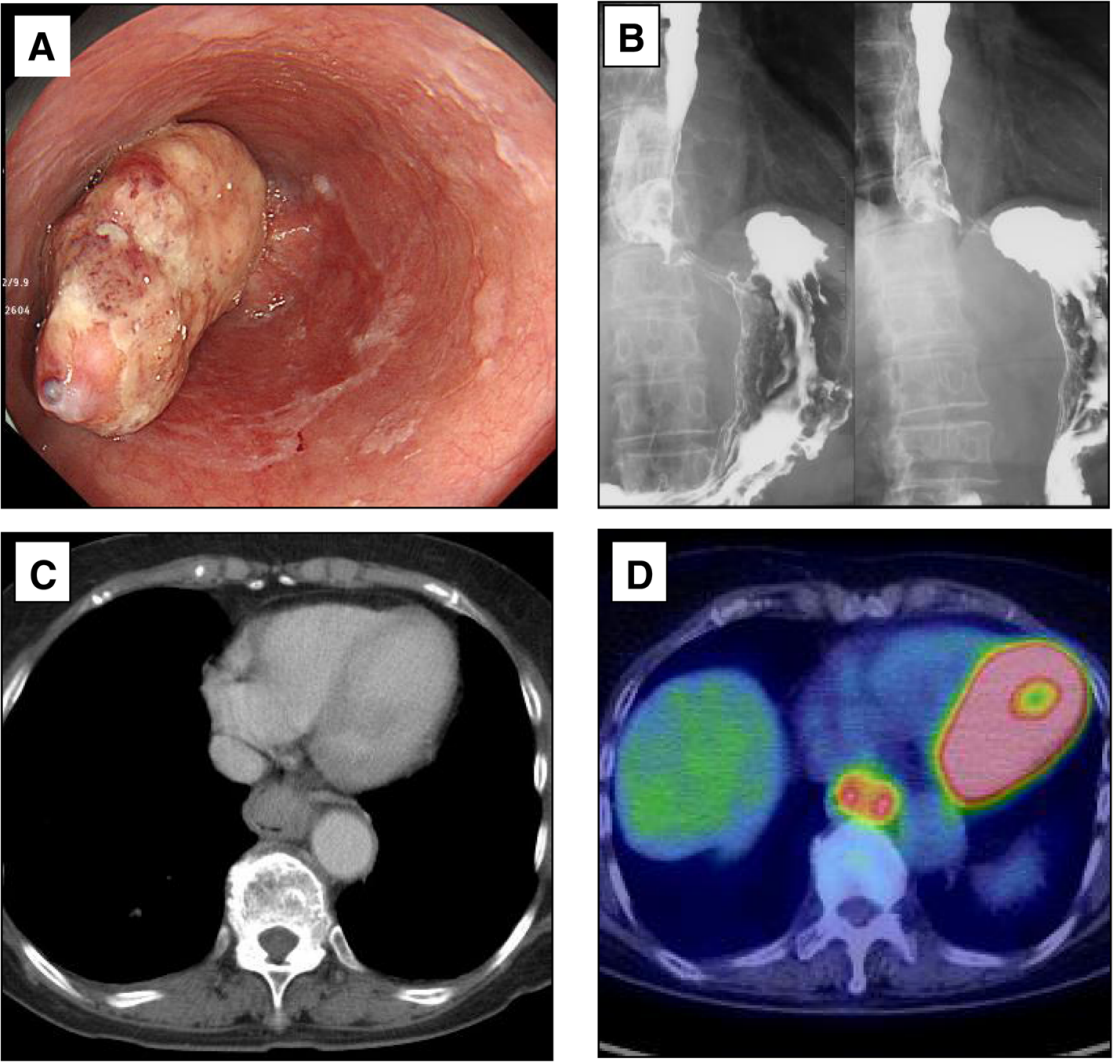
**a** Upper gastrointestinal endoscopy showed a type 1 tumor in the lower thoracic esophagus. **b** Constructed radiography showed the main tumor lesion on the left antero-lateral esophageal wall. **c** Contrast-enhanced CT showed that this tumor lesion measured 3 cm and occupied the esophageal lumen. **d** Positron emission tomography CT showed an uptake in the lower thoracic esophagus and no findings of lymph metastasis

An 86-year-old woman was admitted to our hospital with symptoms of dysphagia. Upper gastrointestinal endoscopy showed an elevated lesion 33–36 cm from an incisor tooth accompanied by ulcers at the center of lesion, which was located in the lower thoracic esophagus (Fig. 1a). Another submucosal tumor located at the anal site of the lower thoracic esophagus was considered intramural metastasis. Esophagography showed the main tumor lesion on the left antero-lateral wall of the lower esophagus and a submucosal tumor on the other side causing constriction of the esophagus. The main lesion had good extension on its basal part, indicating that the depth of invasion was the submucosal level (Fig. 1b). Contrast-enhanced computed tomography (CT) showed the protruded tumor lesion to be 3 cm in size, with no findings of lymph node or distant metastasis (Fig. 1c). Positron emission tomography-CT (PET-CT) showed an increased uptake of fluorodeoxyglucose (^18^F-FDG) in the lower thoracic esophagus and no findings of lymph metastasis (Fig. 1d). A blood test showed that tumor markers, such as carcinoembryonic antigen (CEA) and squamous cell carcinoma associated antigen (SCC), were not elevated. A pathological examination of an endoscopic biopsy revealed moderately to poorly differentiated squamous cell carcinoma. Based on these preoperative analyses, the patient was diagnosed with cT2N0M0, cStageII esophageal squamous cell carcinoma.

Because the patient was elderly and had a poor performance status (PS 2), she did not undergo preoperative therapy, postoperative therapy, or lymph node dissection of the superior to mid-mediastinum regions. We instead performed thoracoscopy- and laparoscopy-assisted subtotal esophagectomy and reconstruction with the gastric tube. Under thoracoscopy and laparoscopy, we performed subtotal esophagectomy and lymphadenectomy, and reconstruction was performed through the retrosternal route. The total operation time was 377 min, and intraoperative blood loss was 105 ml. Oral diet was started 11 days after the operation, and the patient was transferred to another hospital for rehabilitation on day 25 in a good general condition.

**Fig. 2:**
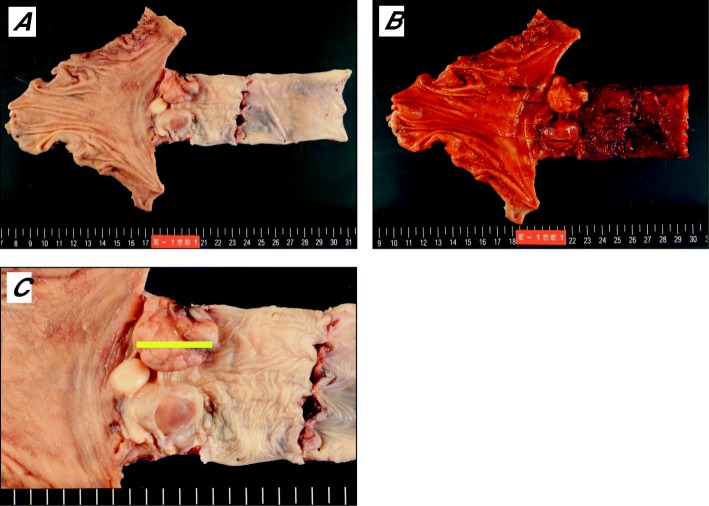
Macroscopic findings. **a**, **b** There were three tumors at the site of the lower thoracic esophagus and gastroesophageal junction, the biggest tumor of which measured 58 × 52 mm; none were stained with Lugol or showed the deposition of melanocytes. **c** Magnified image of the tumors

**Fig. 3:**
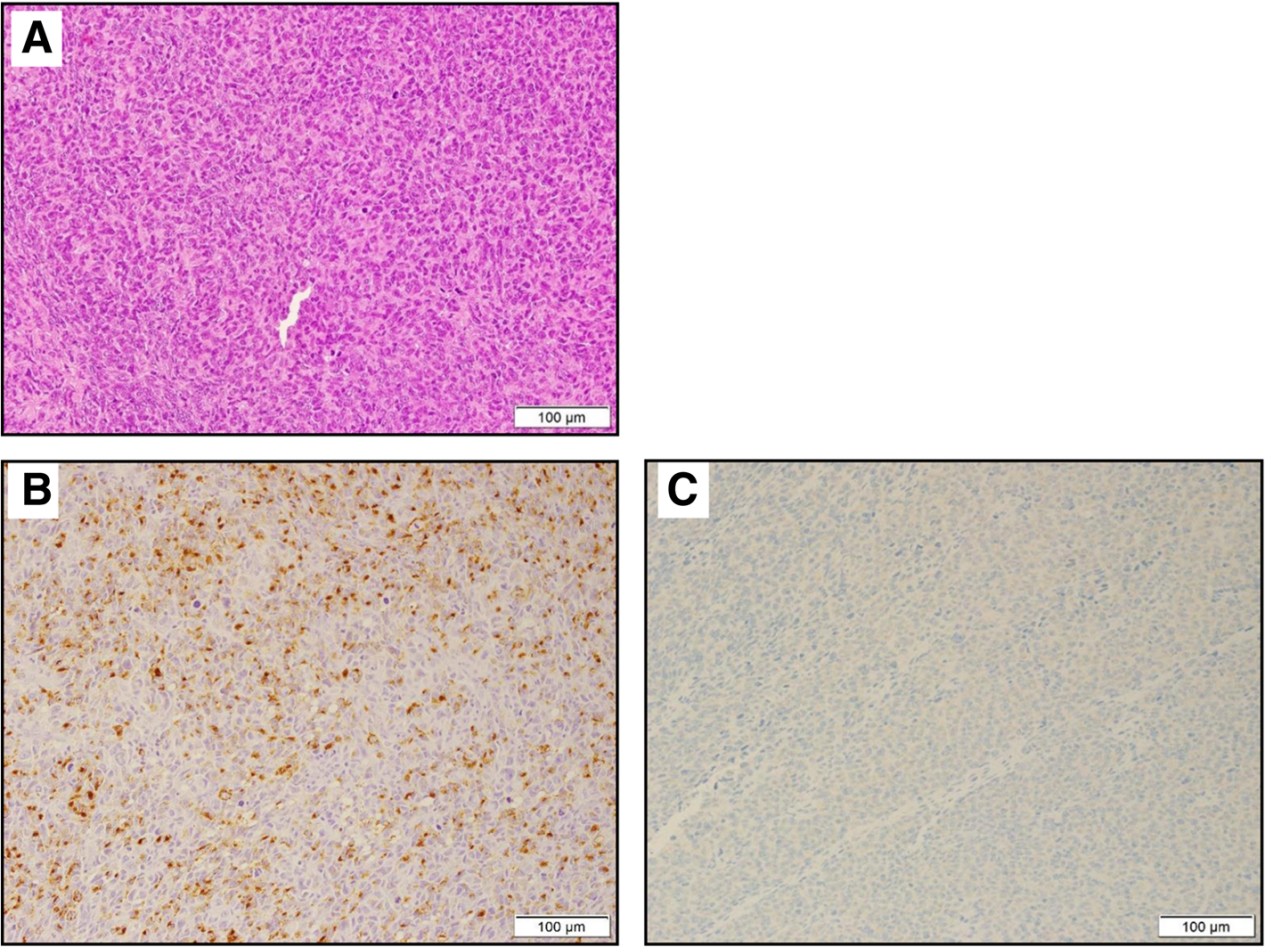
**a** Histopathological findings at the section, which is located on the yellow line in Fig. 2c (hematoxylin and eosin staining). The tumors were located at the submucosa and exhibited hyperplasia-like epithelioid cells but no melanocytes. Histopathological findings (immunohistochemical staining). **b** The tumors were diffusely positive for HMB45. **c** The tumors were partially positive for Melan-A

Three tumors were found in the resected specimen; the biggest tumor was 58 × 52 mm in size, and none were stained with Lugol or showed deposition of melanocytes (Fig. 2a–c). A pathological examination showed that the tumors were located at the mucosa and submucosa of the esophageal wall and were composed of atypical epithelioid cells in a sheeted pattern with necrosis and spindle-shaped cells in a haphazard pattern; however, no melanocytes were observed. Immunohistochemically, atypical epithelioid cells were positive focally for S-100 and HMB45 and partially for Melan-A (Fig. 3), and spindle-shaped cells were positive focally for these markers. However, all of them were negative for almost all of the epithelial markers. We thus decided on a diagnosis of amelanotic type PMME. Although lymph node metastasis at the paracardial lymph nodes (No. 2) was detected, a CT scan performed at 12 months after surgery showed no findings of recurrence.

## Discussion

PMME is a rare disease, accounting for 0.5% of all noncutaneous melanomas with an estimated incidence of 0.0036 cases per million/year [1, 3–5]. The prognosis of PMME is extremely poor. In most cases, the patients are usually diagnosed at a late stage, and 30–40% of them have metastases at the same time. Gao reported that the median survival time of patients with PMME is 18.1 months, and the 1- and 5-year survival rates are 51% and 10%, respectively [6]. Primary amelanotic malignant melanoma, which we experienced in this case, has been reported in only 19 cases from 1996 to 2018 (Table 1) [2, 7–18]. Amelanotic type is especially uncommon among PMME, and its prognosis is also poor.

**Table 1:** Search results for case reports of amelanotic malignant melanoma of the esophagus between 1996 and 2018 [2, 7–18]

Reference	Age/sex Tumor size	Endoscopic findings	Location	TNM stage	Preoperative diagnosis	Treatment	Prognosis
Lee et al. [15]	47/M 3 cm	Polypoid tumor	Lower esophagus	n.a.	n.a.	Surgery	Died 4 months after surgery
Lee et al. [15]	39/F 1.5 cm	Polypoid and ulcerated tumor	Lower esophagus	n.a.	Poorly-differentiated carcinoma	Surgery	No recurrent 4 months from surgery
Suzuki et al. [8]	58/M 6.5 cm	Ulcerated tumor	Middle esophagus	T3N1M0, III	Squamous cell carcinoma	Surgery, CT, IT and ET	Alive 53 months after surgery(recurrence at 11 months)
Heidemann et al. [7]	75/M 8 cm	Ulcerated tumor	Middle and distal esophagus	n.a.	n.a.	Surgery	Recurrent at 7 months
Stringa et al. [10]	59/M 7.5 cm	Polypoid and ulcerated tumor	Lower third of the esophagus	n.a.	n.a.	Surgery	Disseminated 14 months after surgery
De Smione et al. [9]	58/F 3 cm	Polypoid tumor	Middle esophagus	pT1N0M0, IA	Squamous cell carcinoma	Surgery	Died 16 months after surgery
Wang et al. [16]	n.a. n.a.	n.a.	n.a.	n.a.	n.a.	n.a.	n.a.
Kranzfelder et al. [11]	57/F 7 cm	Ulcerated tumor	Distal esophagus	n.a.	PMME	Surgery and RT	Died 4 months after sugery
Terada et al. [12]	87/F n.a.	Ulcerated tumor	Distal esophagus	n.a.	n.a.	CT and RT	Died 12 months after first presentation
Terada et al. [12]	56/M n.a.	Polypoid tumor	Middle esophagus	n.a.	n.a.	CT and RT	Died 7 months after first presentation
Carr-Locke et al. [18]	66/F 3.5 cm	Polypoid and ulcerated tumor	distal esophagus	n.a.	n.a.	Surgery	Alive 2 months after surgery
Lohmann et al. [13]	63/M 3.6 cm	Sessile tumor	Middle esophagus	n.a.	n.a.	Surgery and CT	Died 12 months after diagnosis
Lohmann et al. [13]	67/F 2.2 cm	Polypoid and ulcerated tumor	Middle esophagus	n.a.	n.a.	Surgery	Alive 60 months after surgery
Lohmann et al. [13]	72/M n.a.	Polypoid tumor	Middle esophagus	n.a.	n.a.	Laser ablation	Died 13 months after diagnosis
Lohmann et al. [13]	70/F 10 cm	Sessile and ulcerated tumor	Distal esophagus	n.a.	n.a.	Surgery	Died 2 months after diagnosis
Lohmann et al. [13]	58/F 11 cm	Polypoid and ulcerated tumor	Distal esophagus	n.a.	n.a.	Surgery	Died 4 months after diagnosis
Ramaswamy et al. [17]	24/F 4.3 cm	Polypoid tumor	Cervical esophagus	n.a.	n.a.	Surgery and CT	Died 8 months after first presentation
Hirayama et al. [14]	77/F 2 cm	Polypoid tumor	Middle esophagus	n.a.	PMME	Surgery	No recurence 3 year after surgery
Kobayashi et al. [2]	68/M 2 cm	Polypoid tumor	Middle esophagus	T1bN0M0, IA	Malignant tumor	Surgery	No recurrence 1.5 year after surgery
Current case	86/F 3 cm	Polypoid tumor	Distal esophagus	T2N0M0, II	Squamous cell carcinoma	Surgery	No recurrent finding 12 months from surgery

This article is included in the review of Bisceglia et al. [13]. *M* male, *F* female, *n.a*. not available, *CT* chemotherapy, *RT* radiation therapy, *ET* endocrine therapy, *IT* immunotherapy

One of the reasons for the poor prognosis of amelanotic type is the difficulty of making an early, correct diagnosis. Several factors are implicated in this difficulty. First, the diagnostic criteria for PMME are not easy to apply for a preoperative diagnosis. The Diagnostic Criteria for PMME were defined by Allen and Spitz as follows: (i) a typical histological pattern of melanoma, with melanin granules inside the tumor cells, and (ii) an origin in an area of junctional activity in the squamous epithelium. Junctional activity is defined as melanocytic proliferation in the junctional zone between the dermis and epidermis with its derivatives. In other words, the tumor cells are spread horizontally in the basal layer of the esophageal epithelium. These findings and the presence of in situ melanoma without a history of cutaneous melanoma lead to the absolute diagnosis of PMME [2, 10, 19, 20]. In our case, we recognized junctional activity on surgical specimens but noted no such activity on an endoscopic biopsy sample. It is thus not easy to reach a definitive diagnosis using these criteria because endoscopic biopsy tissue samples tend to be too small to confirm the structure of junctional activity. Second, the findings on an endoscopic examination are not sufficient to make a diagnosis. A black tone is a well-known endoscopic characteristic of PMME, but various other colors, such as purple and brown, are often observed in 10–25% of PMME tumors, depending on the melanin quantity [21, 22]. Amelanotic type produces no melanin pigments, so a preoperative diagnosis is often difficult. It is important to suspect PMME when a black or brown mass is observed in the esophagus. Finally, a biopsy is limited in its ability to support an accurate diagnosis. While a biopsy can aid in the diagnosis of PMME, its accuracy is only approximately 80%. Furthermore, 20–50% of patients are misdiagnosed with poorly differentiated carcinoma, especially in cases of amelanotic melanoma, because of the marked variability in the histological appearance.

The diagnosis of PMME by an endoscopic biopsy is extremely difficult for the following reasons: some tumors are amelanotic and do not contain melanin granules that are detectable by microscopy; melanocytes tend to concentrate in foci and so may be missed by endoscopic biopsy; and the primary esophageal melanoma is often covered by normal squamous epithelium [20, 23]. Therefore, immunohistochemistry (IHC) investigations are useful for obtaining an accurate diagnosis.

However, histology and IHC alone have limitations due to the range of differential diagnoses for PMME, especially for tumors with few or no melanin granules. S-100 protein was originally used to diagnose melanoma [24]. Subsequently, HMB-45 was found to be more specific for melanoma, as it indicated active melanosome formation [25]. Melan-A is another immunohistochemical marker that was found to be positive in a small percentage of HMB-45-negative melanomas [26]. In our review of the literature, the rate of positivity with cytokeratin was reported to be 7% [27]. S-100 therefore seems to be the most sensitive marker for melanoma, while HMB-45 and Melan-A demonstrate relatively good specificity but not as good sensitivity as S-100 [28]. The combination of these antibodies may improve the accuracy of the diagnosis.

It is therefore important to keep PMME in mind when endoscopy shows an esophageal tumor with uncommon findings, especially a tumor with an uncommon color and conduct comprehensive examinations in order to make an accurate diagnosis.

Despite the poor prognosis, there is no consensus for the standard management of PMME of amelanotic type, because it is a rare disease. Surgery is the most common treatment method for PMME, and the benefits of chemotherapy are unclear. However, some authors have reported the effectiveness of neoadjuvant and adjuvant chemotherapy [29]. Chemotherapy was also reported to be beneficial for recurrent cases [30]. Other authors have noted that immune check-point inhibitors, such as anti-CTLA-4 and anti-PD-1 antibodies, benefit patients with advanced PMME [30, 31]. In the accumulated reports, although no significant correlation has been noted between the treatment and prognosis, one patient reported by Suzuki et al. who received neoadjuvant chemotherapy, adjuvant chemotherapy, and immunotherapy after surgery showed a long-term survival [8]. Adjuvant chemotherapy and immunotherapy may be of benefit to patients with PMME; however, further studies will be needed to prove their efficacies.

## Conclusion

In conclusion, we encountered a case of amelanotic type PMME, and the patient remains alive at 1 year after surgery without relapse. The diagnosis of PMME should be based on a combination of findings from a morphological examination, pathological examination, and immunohistochemistry, as PMME sometimes lacks melanin granules inside the tumor cells. It is important to cite PMME as a differential diagnosis of esophageal tumors when in doubt clinically, as the preoperative accurate diagnosis of PMME is difficult, especially for amelanotic type. Improving the accuracy of the diagnosis will ensure that appropriate treatment is provided for patients with PMME.

## Abbreviations

^18^F-FDG: Fluorodeoxyglucose
CEA: Carcinoembryonic antigen
CT: Contrast-enhanced computed tomography
IHC: Immunohistochemistry
PET-CT: Positron emission tomography-CT
PMME: Primary malignant melanoma of the esophagus
SCC: Squamous cell carcinoma-associated antigen

## References

[1] Caldwell CB, Bains MS, Burt M (1991). Unusual malignant neoplasms of the esophagus. Oat cell carcinoma, melanoma, and sarcoma. J Thorac Cardiovasc Surg.

[2] Kobayashi J, Fujimoto D, Murakami M (2018). A report of amelanotic malignant melanoma of the esophagus diagnosed appropriately with novel markers: a case report. Oncol Lett.

[3] Kido T, Morishima H, Nakahara M (2000). Early stage primary malignant melanoma of the esophagus. Gastrointest Endosc.

[4] Mikami T, Fukuda S, Shimoyama T (2001). A case of early-stage primary malignant melanoma of the esophagus. Gastrointest Endosc.

[5] Thrift AP (2016). The epidemic of oesophageal carcinoma: where are we now?. Cancer Epidemiol.

[6] Gao S, Li J, Feng X (2016). Characteristics and surgical outcomes for primary malignant melanoma of the esophagus. Sci Rep.

[7] Heidemann J, Lebiedz P, Herbst H (2005). Amelanotic malignant melanoma of the esophagus: case report. Z Gastroenterol.

[8] Suzuki Y, Aoyama N, Minamide J (2005). Amelanotic malignant melanoma of the esophagus: report of a patient with recurrence successfully treated with chemoendocrine therapy. Int J Clin Oncol.

[9] De Simone P, Gelin M, El Nakadi I (2006). Amelanotic malignant melanoma of the esophagus. Report of a case. Minerva Chir.

[10] Stringa O, Valdez R, Beguerie JR (2006). Primary amelanotic melanoma of the esophagus. Int J Dermatol.

[11] Kranzfelder M, Seidl S, Dobritz M, Brucher BL (2008). Amelanotic esophageal malignant melanoma: case report and short review of the literature. Case Rep Gastroenterol.

[12] Terada T (2009). Amelanotic malignant melanoma of the esophagus: report of two cases with immunohistochemical and molecular genetic study of KIT and PDGFRA. World J Gastroenterol.

[13] Bisceglia M, Perri F, Tucci A (2011). Primary malignant melanoma of the esophagus: a clinicopathologic study of a case with comprehensive literature review. Adv Anat Pathol.

[14] Hirayama Y, Masahiro T, Tanaka T (2017). Slow-growing amelanotic malignant melanoma of the esophagus with long survival: a case report and review of the literature. Endosc Int Open.

[15] Lee SH, Park SH, Kim HG, Kim CB (1998). Primary malignant melanoma of the esophagus. Yonsei Med J.

[16] Wang S, Thamboo TP, Nga M-E (2008). C-kit positive amelanotic melanoma of the oesophagus: a potential diagnostic pitfall. Pathology.

[17] Ramaswamy B, Bhandarkar AM, Venkitachalam S, Trivedi S. Amelanotic malignant melanoma of the cervical oesophagus. BMJ Case Rep. 2014. 10.1136/bcr-2014-204182 PMC3987212 24729119

[18] Carr-Locke DL, Mehra K, Kumar S (2009). Primary amelanotic malignant melanoma of the esophagus.

[19] Levene A (1980). On the histological diagnosis and prognosis of malignant melanoma. J Clin Pathol.

[20] Iwanuma Y, Tomita N, Amano T (2012). Current status of primary malignant melanoma of the esophagus: clinical features, pathology, management and prognosis. J Gastroenterol.

[21] Joob AW, Haines GK, Kies MS, Shields TW (1995). Primary malignant melanoma of the esophagus. Ann Thorac Surg.

[22] Taniyama K, Suzuki H, Sakuramachi S (1990). Amelanotic malignant melanoma of the esophagus: case report and review of the literature. Jpn J Clin Oncol.

[23] Sabanathan S, Eng J, Pradhan GN (1989). Primary malignant melanoma of the esophagus. Am J Gastroenterol.

[24] Volpin E, Sauvanet A, Couvelard A, Belghiti J (2002). Primary malignant melanoma of the esophagus: a case report and review of the literature. Dis Esophagus.

[25] Batsakis JG, Suarez P (2000). Mucosal melanomas: a review. Adv Anat Pathol.

[26] Ron E (1998). Ionizing radiation and cancer risk: evidence from epidemiology. Radiat Res.

[27] Micke O, Schafer U, Glashorster M (1999). Radiation-induced esophageal carcinoma 30 years after mediastinal irradiation: case report and review of the literature. Jpn J Clin Oncol.

[28] Imai S, Suzuki A, Yamamoto Y (2017). Primary malignant melanoma of esophagus following chemoradiotherapy for esophageal squamous cell carcinoma: report of a case. Clin J Gastroenterol.

[29] Yu H, Huang XY, Li Y (2011). Primary malignant melanoma of the esophagus: a study of clinical features, pathology, management and prognosis. Dis Esophagus.

[30] Harada K, Mine S, Yamada K (2016). Long-term outcome of esophagectomy for primary malignant melanoma of the esophagus: a single-institute retrospective analysis. Dis Esophagus.

[31] Wang S, Tachimori Y, Hokamura N (2013). Diagnosis and surgical outcomes for primary malignant melanoma of the esophagus: a single-center experience. Ann Thorac Surg.

